# Mesocotyl Elongation is Essential for Seedling Emergence Under Deep-Seeding Condition in Rice

**DOI:** 10.1186/s12284-017-0173-2

**Published:** 2017-07-14

**Authors:** Hyun-Sook Lee, Kazuhiro Sasaki, Ju-Won Kang, Tadashi Sato, Won-Yong Song, Sang-Nag Ahn

**Affiliations:** 1 College of Agriculture and Life Sciences, Chungnam National University, Daejeon, 305-764 South Korea; 2 Graduate School of Life Sciences, Tohoku University, 2-1-1 Katahira, Aoba-ku, Sendai, 980-8577 Japan; 3 Institute for Sustainable Agro-ecosystem Services, Graduate School of Agricultural and Life Sciences, The University of Tokyo, 1-1-1 Midoricho, Nishitokyo, Tokyo, 188-0002 Japan; 4 Graduate School of Agricultural Science, Tohoku University, 1-1 Amamiya-machi, Tsutsumidori, Aoba-ku, Sendai, Miyagi 980-8555 Japan; 5 POTECH-UZH Cooperative Laboratory, Department of Integrative Bioscience and Biotechnology, Pohang University of Science and Technology, Pohang, 790-784 South Korea

**Keywords:** Rice, Mesocotyl, Coleoptile, Seedling emergence, QTL, CSSLs (chromosome segment substitution lines)

## Abstract

**Background:**

Direct-seeding cultivation by deep-seeding of seeds (drill seeding) is becoming popular due to the scarcity of land and labor. However, poor emergence and inadequate seedling establishment can lead to yield loss in direct-seeding cultivation by deep-sowing. In rice, mesocotyl and coleoptile are primarily responsible for seedling emergence from deeper levels of soil.

**Results:**

Quantitative trait loci (QTLs) for mesocotyl and coleoptile length at 5-cm seeding depth were detected using 98 backcross inbred lines from a cross between Kasalath and Nipponbare. Three QTLs *qMel-1*, *qMel-3*, and *qMel-6* for mesocotyl length were identified on chromosomes 1, 3, and 6, respectively, in two independent replicates. At two QTLs, *qMel-1* and *qMel-3*, the Kasalath alleles increased mesocotyl length, whereas Nipponbare allele increased at *qMel-6*. The Nipponbare alleles at two QTLs (*qCol-3* and *qCol-5*) increased the coleoptile length. Further, seeds of 54 chromosome segment substitution lines (CSSLs) from the cross between Kasalath and Nipponbare sown at 5 cm soil depth showed a significant positive correlation between seedling emergence and mesocotyl elongation (*r* > 0.6, *P* < 0.0001), but not with coleoptile elongation (*r* = 0.05, *P* = 0.7). Seedling emergence of Nipponbare, Kasalath, and the 3 of the 54 CSSLs rapidly decreased with increasing sowing depth. Seedling emergence at seeding depths of 7 and 10 cm was faster in Kasalath and CSSL-5 that harbored the Kasalath alleles across the *qMel-1* and *qMel-3* regions than in the other two CSSLs that contained a single QTL and Nipponbare alleles. CSSL-5 showed the longest mesocotyl among the 3 CSSLs, but no difference in coleoptile length was observed among the 3 CSSLs at seeding depths of 7 and 10 cm.

**Conclusion:**

Variation of mesocotyl elongation was found to be associated with seedling emergence at the seeding depth of 5 cm. To our knowledge, this is the first study performed using CSSLs to detect QTLs for mesocotyl or coleoptile elongation and to determine the effect of mesocotyl elongation on seedling emergence in rice. Our findings provides a foundation for developing rice cultivars that show higher seedling emergence after direct seeding by introgressing QTLs for mesocotyl elongation in rice breeding.

**Electronic supplementary material:**

The online version of this article (doi:10.1186/s12284-017-0173-2) contains supplementary material, which is available to authorized users.

## Background

In Asia, two rice planting methods are used: transplanting and direct seeding. In direct seeding, seeds are sown directly in wet/puddled soil, unpuddled soil, or standing water (Kumar and Ladha 2011). In Asia, approximately 21% of the total rice area is used for direct seeding, and this is expected to increase owing to the scarcity of land, water, and labor (Pandey and Velasco 2005). Rice varieties suitable for direct seeding should include high germination ability, seedling vigor, fast root growth, early tillering ability, and lodging resistance (Lee et al. 2002; Tang 2002). Faster and uniform germination and seedling emergence resulted in more vigorous seedling growth and increasing yield in direct-seeding (Farooq et al. 2006). In direct seeding, deep seeding (drill seeding) is known to reduce damages from wildlife and improve lodging tolerance. However, when seeds are sown deep, the seedlings need to elongate their organs to ensure that the plumule reaches the soil surface. Seedling emergence is an important criterion for determining the actual yield during direct seeding cultivation of rice. The coleoptile (the protective sheath that covers the emerging shoot) and mesocotyl (the structure between the scutellar and coleoptilar nodes in an embryo) of rice are primarily responsible for the emergence of seedlings from deeper soil layers (Turner et al. 1982; Dilday et al. 1990). Seeding depth is also an important factor for dry direct-seeding rice cultivation. Seedling growth for temperate *japonica* cultivars was adversely affected when seeds were sown deeper than the optimum seeding depth of 2–3 cm (Lee et al. 2002). However, shallow seeding can increase the incidences of damage by birds, and plants might suffer lodging after the heading stage. Thus, obtaining information on the optimum seeding depth for direct seeding for diverse germplasm is necessary (Lee et al. 2002).

Previous studies reported that mesocotyl and coleoptile elongation are governed by many genetic and environmental factors (Takahasi 1978; Takahasi 1984). Their elongation shows significant variation across genotypes. Mesocotyl elongation is greater in *indica* rice than in *japonica* rice (Suge 1972; Takahashi 1978; Lee et al. 2012), whereas the coleoptile is longer in *japonica* cultivars than in *indica* cultivars under submerged condition (Takahashi 1978). Among *japonica* cultivars, upland rice generally produced shorter coleoptiles and longer mesocotyls than lowland type (Chang and Vergara 1975).

Quantitative trait loci (QTLs) associated with mesocotyl and coleoptile elongation in rice have been reported. QTLs for mesocotyl elongation were detected using various segregating populations from interspecific or intrasubspecific crosses (Katsuta-Seki et al. 1996; Cai and Morishima 2002; Cao et al. 2002; Huang et al. 2010; Lee et al. 2012). Katsuta-Seki et al. (1996) reported 3 QTLs for mesocotyl elongation on chromosomes 3, 6, and 11 by using F3 populations from the cross between 2 *indica* cultivars grown in glass tubes. Cai and Morishima (2002) detected 11 QTLs for mesocotyl length by using 125 recombinant inbred lines (RILs) derived from a cross between an *indica* cultivar and a strain of wild rice. Further, 5 QTLs for mesocotyl length were detectedusing an RIL population from the cross between 2 *japonica* cultivars by using the filter paper method (Huang et al. 2010). Cao et al. (2002) detected 8 QTLs on chromosomes 1, 3, 6, 7, 8, and 12 under moderate and low temperate conditions in a doubled haploid population derived from a cross between a *japonica* cultivar and an *indica* cultivar. In a previous study, we detected 5 QTLs for mesocotyl length on chromosomes 1, 3, 7, 9, and 12 by using agar media and the same backcross inbred line (BIL) populations used in this study (Lee et al. 2012).

QTLs for improving seedling vigor or seedling establishment, including traits for seedling emergence, seed germination, or shoot length, and coleoptile length have been detected (Redoña and Mackill 1996; Zhou et al. 2007; Xie et al. 2014). Redoña and Mackill (1996) detected 2 QTLs for coleoptile length and 5 QTLs for mesocotyl length by using an F2:F3 population derived from a cross between *japonica* cultivar Labelle and *indica* cultivar Black Gora. Further, 5 QTLs for seed vigor traits such as coleoptile length and radicle length and 3 QTLs for seed germination under low and normal temperature conditions were identified using populations derived from a cross between two *indica* cultivars (Xie et al. 2014). In addition, 9 QTLs for seedling vigor traits, including coleoptile emergence, were detected under two field conditions—drained and flooded soil—and 2 QTLs for coleoptile emergence were identified on chromosomes 1 and 3 under flooded soil condition (Zhou et al. 2007).

In this study we aimed to show that mesocotyl elongation is essential to seedling emergence under deep-seeding using chromosome segment substitution lines (CSSLs). Although several studies identified QTLs for mesocotyl or coleoptile elongation, the locations and effects of the detected QTLs varied according to the test methods (Redoña and Mackill 1996; Zhou et al. 2007; Huang et al. 2010; Xie et al. 2014). Furthermore, no study tried to characterize and detect QTLs for mesocotyl under deep-seeding condition in rice. Also, study to analyze the association between mesocotyl elongation and seedling emergence under various soil depth conditions is rare in crop including rice (Chung 2010; Alibu et al. 2011; Lu et al. 2016).

This study attempted to (1) identify QTLs associated with mesocotyl and coleoptile elongation under the deep-seeding soil condition, and (2) to evaluate the effect of mesocotyl elongation on seedling emergence using deep-seeded CSSLs.

## Results

### Phenotypic variation of mesocotyl and coleoptile elongation

**Fig. 1:**
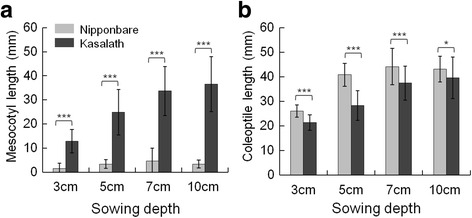
Phenotypic variation of mesocotyl and coleoptile elongation at varying soil depths. **a** Mesocotyl and **b** coleoptile length of Nipponbare and Kasalath under different burial depth in soil; 12 Seeds of Nipponbare and Kasalath were sown at 3 cm, 5 cm, 7 cm, and 10 cm soil depth and incubated at alternate temperatures of 30 °C and 26 °C (14 h/10 h). At 14 days after sowing, the seedling were excavated and length were measured. Bars represent mean of length with SD (*n* = 3). Comparisons of the varieties were made with the ANOVA test. * *P* < 0.05, *** *P* < 0.001

Nipponbare and Kasalath were selected to determine the seeding depth for detecting QTLs controlling mesocotyl and coleoptile lengths. At first, Nipponbare and Kasalath seeds were placed at 5 cm and 7 cm sowing depths based on previous reports (Chung 2010, Alibu et al. 2011). Although Nipponbare and Kasalath seeds completely germinated, some seedlings showed poor growth at 7 cm depth (Additional file 1: Figure S1). Two soil depth conditions, 3 cm (shallower soil depth than 5 cm) and 10 cm (deeper soil depth than 7 cm) were added to measure mesocotyl and coleoptile length at 14 days after sowing (Fig. 1). Kasalath showed exceptionally long mesocotyls than those in Nipponbare at all soil depths. Such difference in mesocotyl length between these two varieties has also been observed using agar media (Lee et al. 2012). In contrast, the coleoptile length was significantly longer in Nipponbare than in Kasalath at all soil depths (*P* < 0.05). Both mesocotyl and coleoptile lengths increased with sowing depth in both the cultivars, but the difference was not significant between 7 and 10 cm sowing depths. The mesocotyl lengths were 24.9 mm (seeding depth, 5 cm), 33.7 mm (7 cm), and 36.5 mm (10 cm) in Kasalath, whereas the coleoptile lengths were 40.8 mm (5 cm), 44.2 mm (7 cm), and 43.2 mm (10 cm) in Nipponbare. These results suggested that the mesocotyl of Kasalath maximally elongates to about 35 mm, and the coleoptile in Nipponbare maximally elongates to about 43 mm, under the experimental conditions used in this study. Therefore, 7 cm seeding depth was considered suitable for QTL analysis of mesocotyl and coleoptile lengths in the BILs. Nevertheless, 5 cm seeding depth was selected for QTL analysis. Because, some seed grown insufficiently after germination hindered the accurate phenotypic evaluation of the traits when planted in 7 cm and 10 cm soil depths (Additional file 1: Figure S1). Mesocotyl and coleoptile length of Nipponbare and Kasalath seedling were measured except insufficiently grown seedling in 7 cm and 10 cm soil depth (Fig. 3c and d).

**Fig. 2:**
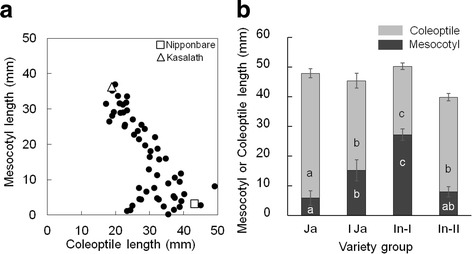
Mesocotyl and coleoptile length for 57 accessions of RDRS (**a**), Comparison of mesocotyl and coleoptile length in 4 rice variety groups (**b**) Japonica (Ja), Tropical Japonica (TJa), Indica-I (In-I), and Indica-II (In-II); Seeds were sown at 5 cm soil depth and incubated at alternate temperatures of 30 °C and 26 °C (14 h/10 h). At 14 days after sowing, the seedling were excavated and length were measured. **b** Means with different letters in the same row indicate significant differences according to the Duncan multiple range test (*P* < 0.001). *Bars* represent mean of length with SE

Fifty-seven accessions from a rice diversity research set of germplasm (RDRS) were evaluated for mesocotyl and coleoptile length to know the association between two traits and also to clarify whether 5 cm seeding depth was a proper condition for analyzing phenotypic variation. This collection carries 91% of the alleles identified in the 332 original rice varieties and covers most of the range of variation in several agro-morphological traits from original varieties (Kojima et al. 2005). Phenotypic variation in mesocotyl and coleoptile lengths in 57 RDRS accessions is shown in Fig. 2 and Additional file 2: Table S1. The rice accessions showed large variations for both mesocotyl and coleoptile lengths. The mesocotyl length of the 57 accessions ranged from 0.3 mm to 37 mm, whereas the coleoptile length ranged from 17.3 mm to 49.2 mm (Fig. 2a). The mesocotyl length showed a significant negative correlation with the coleoptile length (*r* = −0.73, *P* < 0.001). Moreover, comparison of mesocotyl and coleoptile elongation among the four groups—*japonica*, tropical *japonica*, *indica*-I, and *indica*-II, classified by Kojima et al. (2005) (Uga et al. 2009; http://www.gene.affrc.go.jp/databases-core_collections_wr.php)—showed that the difference among the groups was highly significant for both the traits (*P* < 0.001; Fig. 2b). The *indica*-I group (including Kasalath) and *japonica* group (including Nipponbare) showed the longest mesocotyl (27.2 mm) and coleoptile (41.9 mm), respectively (Fig 2b). Among the accessions, Nipponbare and Kasalath displayed huge difference in mesocotyl and coleoptile length indicating that the BILs from a cross between Nipponbare and Kasalath were suitable for QTL analysis. Also, the BILs were used for mapping QTLs for a wide variety of traits (Lin et al. 1998; Murai et al. 2002; Yamaya et al. 2002). Therefore, 5 cm seeding depth was used to analyze the genetic variation in mesocotyl and coleoptile elongation among the 98 BILs from a cross between Kasalath and Nipponbare.

**Fig. 3:**
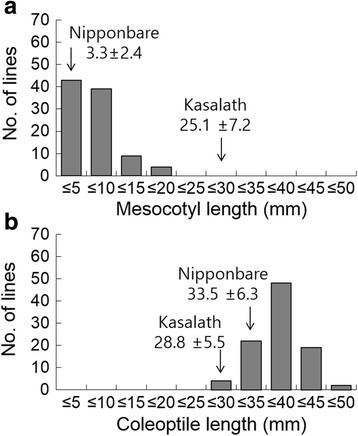
Frequency distribution of the mesocotyl length (**a**) and coleoptile length (**b**) in 98 backcross inbred lines (BILs) derived from Nipponbare/Kasalath at 5 cm soil depth; *Arrowheads* indicate mean values for Nipponbare and Kasalath. 12 seeds of 98 BILs, Nipponbare and Kasalath were sown at 5 cm soil depth and incubated at alternate temperatures of 30 °C and 26 °C (14 h/10 h). At 14 days after sowing, length were measured

Two independent measurements of mesocotyl and coleoptile lengths of the 98 BILs were obtained at the 5 cm seeding depth (Fig. 3). The mesocotyl and coleoptile lengths were not significantly different between the two independent experiments (*r* = 0.79 for mesocotyl, *r* = 0.66 for coleoptile; *P* < 0.001). The mesocotyl lengths of Nipponbare and Kasalath were 3.3 mm and 25.1 mm, respectively, in one replicate (Fig. 3a). In the 98 BILs, the mesocotyl length ranged from 0.7 mm to 19.3 mm, with an average of 6.5 mm. The coleoptile lengths of Nipponbare and Kasalath were 33.5 mm and 28.8 mm, respectively, in one replicate (Fig. 3b.), and the average coleoptile length was 37.1 mm and ranged from 27.6 mm to 47.2 mm in the BILs. Mesocotyl length showed a highly significant negative correlation with coleoptiles length in both the replicates (*r* = −0.37, *P* < 0.001 in 1st replicate and *r* = −0.20, *P* < 0.05 in 2nd replicate). In BILs, no single line had longer mesocotyl than Kasalath whereas some transgressive lines showing longer coleoptile length than Nipponbare were observed. Lack of transgressive lines for mesocotyl length is possibly due to the finding that Kasalath has major mesocotyl increasing QTLs, *qMel-1* and *qMel-3* whereas Nipponbare has a minor QTL whose effect was negligible in BILs. For coleoptile length, it is possible that Kasalath might possess a few QTL with positive effects on coleoptile elongation although two minor QTLs were detected with increasing effects from Nipponbare due to small population size of BIL, and gene interactions. This result is consistent with that of the previous studies (Redoña and Mackill 1996; Xie et al. 2014).

### QTL analysis for deep-seeding tolerance-related traits

QTL was detected based on logarithm of odds ratio (LOD) thresholds after the permutation test. In all, 5 QTLs, including 3 for mesocotyl length and 2 for coleoptile length, were detected at the 5 cm seeding depth (Table 1, Additional file 3: Figure S2). The three QTLs (qMel*-1*, *qMel-3*, and *qMel-6*) for mesocotyl length were mapped near the markers—R2414, R1618, and R2123 on chromosomes 1, 3, and 6, respectively. The *qMel-3* QTL was detected in both the replicates (Rep1 and 2), and the Kasalath alleles at two QTL loci (qMel*-1* and *qMel-3*) contributed to an increase in mesocotyl length. The QTL *qMel-1* accounted for 13.89% and *qMel-3* for 37.56% and 26.97% of phenotypic variance. These QTLs were detected even when the varieties were cultured in agar (Lee et al. 2012). Two QTLs, *qCol-3* and *qCol-5* for coleoptile length were mapped near the markers, C25 and C597 on chromosomes 3 and 5, respectively, in one replicate (Rep 1). In the other replicate (Rep 2), no QTLs for coleoptile length were detected. The two QTLs, *qCol-3* and *qCol-5* accounted for 11.8 and 12.0% of phenotypic variance, respectively. Nipponbare alleles at the two QTL loci contributed to an increase in the coleoptile length. Interestingly, *qMel-3* and *qCol-3* showed loose linkage suggesting a possibility of combining two QTLs via marker-assisted selection.

**Table 1:** Characteristics of QTLs for mesocotyl and coleoptile length in 98 BILs

Locus ^a^	Chr.	Nearest marker	Position	Replication ^b^	LOD score ^c^	R2 (%) ^d^	Additive effect	Positive allele ^e^
*qMel-1*	1	R2414	39.6- Mb	Rep 2	3.44	13.89	4.34	K
*qMel-3*	3	R1618	30.7-Mb	Rep 1	12.09	37.56	6.41	K
		R1618	30.7-Mb	Rep 2	7.18	26.97	6.46	K
*qMel-6*	6	R2123	11.68-Mb	Rep 2	2.81	8.28	3.89	N
*qCol-3*	3	C25	3.97-Mb	Rep 1	3.06	11.8	2.96	N
*qCol-5*	5	C597	0.26-Mb	Rep 1	3.20	12.0	2.70	N

^a^ QTLs were designated as “*qMel* chromosome number” and “*qCol* chromosome number”

^b^ QTLs were detected under soil conditions in two replications, respectively

^c^ Maximum LOD score over threshold significance level at *P* < 0.05

^d^ Proportion of the phenotypic variation explained by the nearest marker of QTL

^e^ K and N respectively represent the positive effects of QTL contributed by Kasalath and Nipponbare alleles

### Effect of mesocotyl elongation on seedling emergence

**Fig. 4:**
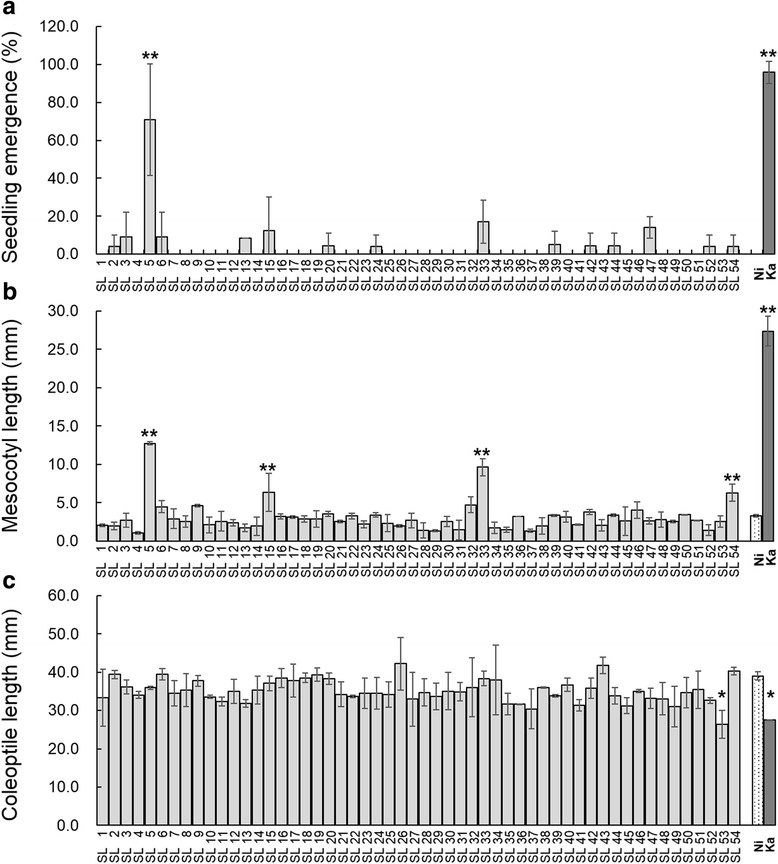
Seedling emergence percentage at 6 days after sowing (**a**), mesocotyl (**b**) and coleoptile length (**c**) of 54 CSSLs (SL); 12 seeds of 54 CSSLs, Nipponbare and Kasalath were sown at 5 cm soil depth and incubated and the numbers of emerged seedling from the soil surface were counted. At 14 days after sowing, the length were measured. Each column represents emergence percentage or mean of length ± SE * *P* < 0.05 and **P < 0.01 versus Nipponbare (Dunnett’s multiple comparison test, two replicates)

In all, 54 CSSLs were used for evaluating the effect of QTLs for mesocotyl or coleoptile length on seedling emergence at the 5 cm seeding depth (Fig. 4). Each CSSL carries a single or a few Kasalath segments in the near-isogenic background of Nipponbare. Seedlings started to emerge at 6 days after sowing, when the analysis was conducted.

A positive correlation was found between mesocotyl length and seedling emergence at 6 days after sowing (*r* = 0.8, *P* < 0.0001), whereas no significant correlation was noted between coleoptile length and seedling emergence (*P* > 0.5). This result suggests that seedling emergence is dependent on mesocotyl elongation.

All lines except 5 lines were not significantly different from Nipponbare in mesocotyl and coleoptile lengths (Fig. 4). Four lines, CSSL-5, CSSL-15, CSSL33 and CSSL-54 showed significantly longer mesocotyl length than Nipponbare and the other CSSLs (Fig. 4b). CSSL-5 showed the highest emergence percentage (70%) among CSSLs followed by CSSL-33 (17%) and CSSL-15 (12.5%). (Fig. 4a). CSSL-5 possessed Kasalath segments across *qMel-1* and *qMel-3* and across only *qMel-3* in CSSL-15. On the other hand, CSSL-33 possessed the Kasalath segments across *qMel-3* and *qMel-7*. The *qMel-7* QTL was not detected in this study whereas it was observed in the agar condition (Lee et al. 2012). Coleoptile length of Kasalath and CSSL-53 was significantly shorter than Nipponbare and the other CSSLs, but the emergence percentage of Kasalath (87.5%) was the highest (Fig. 4a and C). This result suggests that seedling emergence is dependent on mesocotyl elongation.

**Fig. 5:**
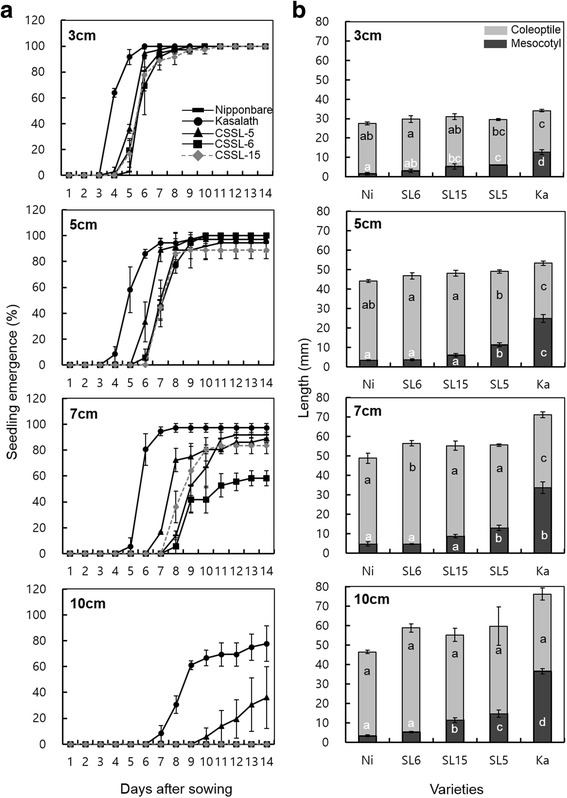
Time course of seedling emergence (**a**) and mesocotyl and coleoptile length (**b**) of 3 CSSLs, Nipponbare and Kasalath in four soil burial depth; (**a**) Lines and bars represent the mean with SE of 5 lines, Nipponbare(Ni), Kasalath (Ka), CSSL-6 (SL6), CSSL-15 (SL15), and CSSL-5 (SL5). Seeds were sown at 3 cm, 5 cm, 7 cm, and 10 cm soil depth and the number of emerged seedling form soil surface were counted daily up to 14 days after sowing. **b** At 14 days after sowing, length of mesocotyl and coleoptile were measured. Means of length ± SE (*n* = 3) with different letters in the same row indicate significant differences according to the Duncan multiple range test (*P* < 0.05)

In addition, we tried to confirm the effect of mesocotyl length on seedling emergence using 3 CSSLs (Fig. 5). Lengths of mesocotyl and coleoptile and seedling emergence were investigated in 3 CSSLs, possessing the Kasalath segments across the *qMel-1* (CSSL-6) or *qMel-3* (CSSL-15) regions and both (CSSL-5). Seedlings of all genotypes emerged over 90% at 3 cm and 5 cm seeding depths from 8 to 9 days after sowing (Fig. 5a). At 7-cm seeding depth, Kasalath and CSSL-5 showed faster emergence than the other genotypes. At 10-cm seeding depth, no seedlings of Nipponbare, CSSL-6, and CSSL-15 emerged even 14 days after sowing, whereas Kasalath and CSSL-5 seedlings emerged and showed 78 and 36% seedling emergence after 14 days, respectively (Fig. 5a). The coleoptile length of CSSL-5 were not significantly different from those of Nipponbare or the other two CSSLs at all seeding depth. On the contrary, the mesocotyl length of CSSL-5 showed the longest length among 3 CSSLs at 5-cm, 7-cm and 10-cm seeding depth (Fig. 5b). These results indicate that a significant relationship exists between mesocotyl elongation and seedling emergence in deep-seeded plants.

Additionally, these results also show that the 2 QTLs, *qMel-1* and *qMel-3* act additively in distinct or complementary pathways in controlling mesocotyl elongation under this study condition.

## Discussion

In rice, seeding depth for direct seeding is an important factor for ensuring seedling establishment. Lee et al. (2002) recommended a 2–3-cm sowing depth for *japonica* rice. When the seeding depth is >3 cm, seedling emergence was markedly delayed (Kawatei et al. 1963; Murai et al. 1995; Luo et al. 2007; Chung 2010; Alibu et al. 2012). Moreover, when seeds were sown deeper than 5 cm, the first leaf developed under the soil surface, and seedling establishment decreased remarkably (Chung 2010). Similar result was obtained in this study: seedling emergence speed and percentage at 7 cm and 10 cm soil depths were lower than those at the seeding depth of 3 cm in all the genotypes (Fig. 5). Although 3-cm seeding depth might be optimal for direct seeding based on these results, many studies have used seeding depths of less than 3 cm, which caused seed desiccation and floating and seedling lodging (Lee et al. 2002, Kumar and Ladha 2011). Genotype differences for seedling emergence and coleoptile length were more pronounced at 5 cm than at 3 cm seeding depth (Figs. 1 and 5). The two parents and the 3 CSSLs showed >90% seedling emergence within 7 days when the seeding depth was 3 cm, whereas Kasalath and CSSL-5 showed >90% emergence and the other three lines reached a plateau at 80% emergence at 5 cm seeding depth (Fig. 5). These results indicate that soil depth of 3–5 cm is appropriate for direct seeding of cultivars that show good mesocotyl elongation without affecting seedling establishment. Based on these findings, we selected 5 cm soil depth for screening seedling emergence traits under the deep-seeding condition.

Mesocotyl and coleoptile lengths are directly related with seedling emergence in deep seeding and enhanced mesocotyl or coleoptile elongation is associated with better seedling emergence and establishment (Turner et al. 1982; Murai et al. 1995; Luo et al. 2007; Chung 2010; Alibu et al. 2012). Murai et al. (1995) reported that a relationship exists between seedling emergence ability and lengths of leaf, mesocotyl, coleoptile, and leaf internode using dwarf lines under 7 cm soil depth. Chung (2010) reported positive correlations between the rate of seedling emergence and mesocotyl or coleoptile length at 5 cm seeding depth by using 116 Korean weedy rice accessions. Alibu et al. (2012) suggested that upland rice accessions with long mesocotyl showed better seedling emergence than those with long coleoptile under the dry direct-seeding condition. Lu et al. (2016) showed that long mesocotyl rice accessions had higher emergence rate than that of the short one at 2 and 5-cm sowing depth. In addition to the seeding depth, in dry seeding, the lengths of mesocotyl and coleoptile can be affected by moisture content. Under submergence, coleoptile growth was stimulated, although no increase in mesocotyl length was observed (Takahashi 1978; Alibu et al. 2011). This suggests that mesocotyl and coleoptile elongation is important for seedling emergence depending on the environment. In our study, a positive correlation between mesocotyl length and seedling emergence was observed (Figs. 4 and 5). Seedlings of Kasalath and CSSL-5 that have long mesocotyls emerged considerably faster than those of the other genotypes that have short mesocotyl at the 7 and 10 cm seeding depths (Figs. 4 and 5). At 7 cm seeding depth, seedlings of Kasalath and CSSL-5 emerged over 70% in 7-8 days after sowing, whereas those of the other genotypes and Nipponbare showed 70% emergence at 10–11 days after sowing. This delayed seedling emergence of up to 3–4 days might be unfavorable for seedling establishment and competition with weeds.

Evaluation of RDRS showed genotypic variation of mesocotyl and coleoptile length (Fig. 2). *Japonica* accessions tend to have shorter mesocotyls than *indica*, whereas *japonica* showed the longest coleoptiles among four groups. In this study, *indica*-I accessions had the longest mesocotyls among the four groups, followed by the tropical *japonica* group (Fig. 2b). These results are consistent with the previous findings (Suge 1972; Takahashi 1978, Sato 1987).

QTL analysis was conducted for mesocotyl and coleoptile length of seedlings sown at 5 cm soil depth. Measuring mesocotyl and coleoptile traits in the soil is difficult mainly due to the environmental influence and experimental errors. Several studies attempted to map QTLs for mesocotyl and coleoptile length by using the glass tube method (Katsuta-Seki et al. 1996), slant-board test (Redoña and Mackill 1996), filter paper with distilled water (Huang et al. 2010), agar medium (Lee et al. 2012), and filter paper on agar medium (Xie et al. 2014). However, QTL analysis for mesocotyl or coleoptile elongation at the 5 cm seeding depth by using CSSLs in rice has not yet been performed. We detected 5 QTLs under field condition in two replicates. Interestingly, QTLs for mesocotyl elongation were commonly mapped to chromosomes 1 and 3 for different mapping populations and under different experimental conditions. The QTL *qMel-3* was located on the long arm of chromosome 3, and this QTL interval overlapped with regions of QTLs reported in previous studies (Katsuta-Seki et al. 1996; Redoña and Mackill 1996; Cai and Morishima 2002; Cao et al. 2002; Huang et al. 2010). Using genome-wide association study (GWAS) association mapping, Wu et al. (2015) and Lu et al. (2016) identified some candidate genes controlling mesocotyl elongation on chromosome 1 and 3. Coleoptile QTLs were detected on chromosomes 3 and 6 by Redoña and Mackill (1996) and on chromosomes 5, 8, and 11 by Xie et al. (2014). On chromosomes 1 and 3, two QTLs for coleoptile length were identified under flooded soil condition (Zhou et al. 2007). Interestingly, *qCol-3* and *qCol-5* that were detected in the present study did not overlap with the coleoptile length QTLs that was reported previously; this could be attributed to the different genetic materials and test methods used.

Among 54 CSSLs, two lines, CSSL-5 and CSSL-33 showed significantly longer mesocotyl than Nipponbare. CSSL-5 showed the highest emergence ratio (70%) followed by CSSL-33 (17%) (Fig. 4a and b). CSSL-5 and CSSL-33 shared *qMel-3* suggesting that *qMel-3* is a major QTL. In contrast, *qMel-7* is minor and affected by testing methods based on the finding that the *qMel-7* QTL was detected in the agar condition (Lee et al. 2012) but not in the soil condition in this study. However, CSSL-5 seedlings were shorter in mesocotyl length and lower in emergence rate than Kasalath. The results from this study indicate that the *qMel-3* QTL with a high additive effect has the potential to enhance early seedling emergence in combination with the *qMel-1, qMel-7* and other QTLs not detected. Also, information on the interaction among these QTLs will be necessary to understand the genetic control of mesocotyl length. These findings will contribute to our understanding of the genetic control of mesocotyl and coleoptile elongation, and seedling emergence and provides information on QTL regions associated with early seedling emergence for rice breeding programs.

## Conclusions

We showed that the variation in mesocotyl elongation is associated with seedling emergence under the deep-seeding condition. Genetic analysis using BIL and CSSL plants confirmed that two major QTLs, *qMel-1* and *qMel-3* for mesocotyl elongation have the potential to enhance early seedling emergence at 5 cm seeding depth. This is the first report to show the effect of two QTLs for mesocotyl elongation detected both in agar media and deep-seeding soil on seedling emergence using CSSLs using deep-seeding soil condition. (Lee et al. 2012). High-resolution mapping and cloning of these QTLs should be performed to understand the genetic control of mesocotyl elongation to ensure higher seedling emergence and to develop deep-seeding-tolerant rice cultivars for direct seeding by using marker-assisted selection.

## Methods

### Plant materials

Fifty-seven rice accessions selected from the RDRS of germplasm collection were used to detect variation in mesocotyl and coleoptile elongation (*Oryza sativa* L.) (Kojima et al. 2005; Lee et al. 2012; Additional file 2: Table S1). QTLs for mesocotyl and coleoptile elongation were detected and the effect of mesocotyl elongation on seedling emergence was determined by using 98 BILs and 54 CSSLs developed from the backcross of the Nipponbare/Kasalath (Lin et al. 1998). Seeds of BILs and CSSLs were provided by Rice Genome Resource Center (RGRC), Japan (http://www.rgrc.dna.affrc.go.jp/stock.html).

The effect of QTLs for mesocotyl elongation *qMel-1* and *qMel-3* on seedling emergence was determined by selecting 3 CSSLs—CSSL-6, CSSL-15, and CSSL-5 (Lee et al. 2012). The genotype data of the CSSLs were referred from a database of RGRC (http://www.rgrc.dna.affrc.go.jp/ineNKCSSL54.html). CSSL-6 and CSSL-15 carried the QTLs *qMel-1* and *qMel-3*, respectively. CSSL-6 contained a 34-cM Kasalath segment flanked by restriction fragment length polymorphism (RFLP) markers C86-C112 carrying *qMel-1* on chromosome 1. CSSL-15 contained a 61-cM Kasalath segment flanked by RFLP markers R19–R1925 carrying *qMel-3* on chromosome 3 in the Nipponbare background. CSSL-5 carried both QTLs *qMel-1* and *qMel-3* in the Nipponbare background. In CSSL-5, 2 Kasalath segments on qMel-1 and qMel-3 were introgressed, a 34-cM Kasalath segment near the RFLP makers C1370–2018, including *qMel-1*, 43-cM Kasalath segment near RFLP markers C136–R1925, in the Nipponbare background.

RDRS, BIL, and CSSL plants were grown in the experimental lowland field of the Graduate School of Life Sciences, Tohoku University, Kashimadai, Osaki, Miyagi Prefecture, Japan. The panicles of these plants were harvested at 40–50 days after heading and then dried in a well-ventilated space for 3 months. Dried seeds were collected from the panicle and placed in paper envelopes. All of the seeds were completely germinated in 30 °C imbibitions for 4 days. For maintaining the germinability, the seed envelopes were packed in plastic bags sealed with silica gel and stored at 4 °C in a refrigerator until use.

### Measurement of mesocotyl and coleoptile elongation

Mesocotyl and coleoptile elongation was measured and seedling emergence under the soil condition was evaluated by sowing 12 good-quality seeds from each RDRS accession, BILs, and CSSLs at various soil depths. Seeds of Nipponbare, Kasalath, and 3 CSSLs were buried at a depth of 3, 5, 7, and 10 cm in plastic pots (diameter, 11 cm; height, 15 cm) in soil containing chemical fertilizer (Nursery culture soil No. 3; Mitsui-Toatsu, Japan). For determining phenotype variation in mesocotyl and coleoptile length, seeds of RDRS, 98 BILs and 54 CSSLs were buried in 5 cm soil depth. The plastic pots were kept in plant growth cabinets at alternate temperatures of 30 °C and 26 °C (14 h/10 h). The water level of the soil was maintained 2 cm from the bottom of the pots to maintain adequate soil moisture for germination and seedling growth. At 14 days after sowing, the seedlings were carefully excavated and washed, and their lengths were measured. Seedlings insufficiently grown were excluded from measurement. In this study, measurement was made on seedlings showing 3rd-4th leaves expansions at 14 days after sowing. Mesocotyl (the distance from the basal part of the seminal root to the coleoptilar node) and coleoptile (the distance from the coleoptilar node to the tip of the coleoptile) lengths of each seedling were measured using a ruler. Seedlings that germinated but grew insufficiently were excluded from the length measurements. The numbers of emerged seedlings from the soil surface were counted daily up to 14 days when seedling emergence was thought to be complete. The experiment was performed in a completely randomized design with two or three replicates of each line.

### Genotype data of BILs and QTL analysis

The QTLs controlling mesocotyl and coleoptile elongation in BILs were mapped by using genotype data generated using 245 RFLP markers (http://www.rgrc.dna.affrc.go.jp/ineNKBIL98.html). Linkage analysis of the genotypic data was performed using the Kosambi function of Mapmaker/EXP 3.0 software (Lander et al. 1987). QTL analysis was performed using composite interval mapping (CIM) by using the QTL Cartographer version 2.5 software (Wang et al. 2012). CIM analysis was performed using forward-backward stepwise regression model 6 with a 10-cM window size. The LOD threshold significance level (*P* < 0.05) was determined by computing 1000 permutations. The QTL positions were assigned to the points of maximum LOD score in the target regions. The percentage of total phenotypic variance noted for each QTL was estimated on the basis of the *R* ^*2*^ value.

## Additional files

Additional file 1: Figure S1. Seedling of Nipponbare and Kasalath growing at 5 cm and 7 cm soil depth condition; 50 seeds of Nipponbare and Kasalath were sown at 5 cm and 7 cm soil depth and incubated at alternate temperatures of 30 °C and 26 °C (14 h/10 h). At 21 days after sowing, the seedling were excavated and the length of coleoptile (Col) and mesocotyl (Me) were measured. Arrows indicate mesocotyl. (TIFF 6962 kb)

Additional file 2: Table S1. Variation of the mesocotyl and coleoptile length for 57 rice accessions from RDRS collection. (PDF 73 kb)

Additional file 3: Figure S2. QTL cartographer LOD peak for mesocotyl and coleoptile length at 5 cm soil depth; A QTL Cartographer plot obtained following composite interval mapping (CIM) using 2 replicates (Rep1 and 2). Significance of QTL is indicated by LOD score above the threshold values determined by permutation analysis at a significant level of *P* < 0.05. The graph below shows the additive effects for each of the QTL identified. Marker designations are given at the bottom and the genetic distances (cM) are given above the horizontal line. (TIFF 4914 kb)
