# Supplementary figures and images for: Mesocotyl Elongation is Essential for Seedling Emergence Under Deep-Seeding Condition in Rice
Source: Rice (N Y). 2017 Jul 14;10:32. doi: 10.1186/s12284-017-0173-2 (PMC5511125; doi:10.1186/s12284-017-0173-2)

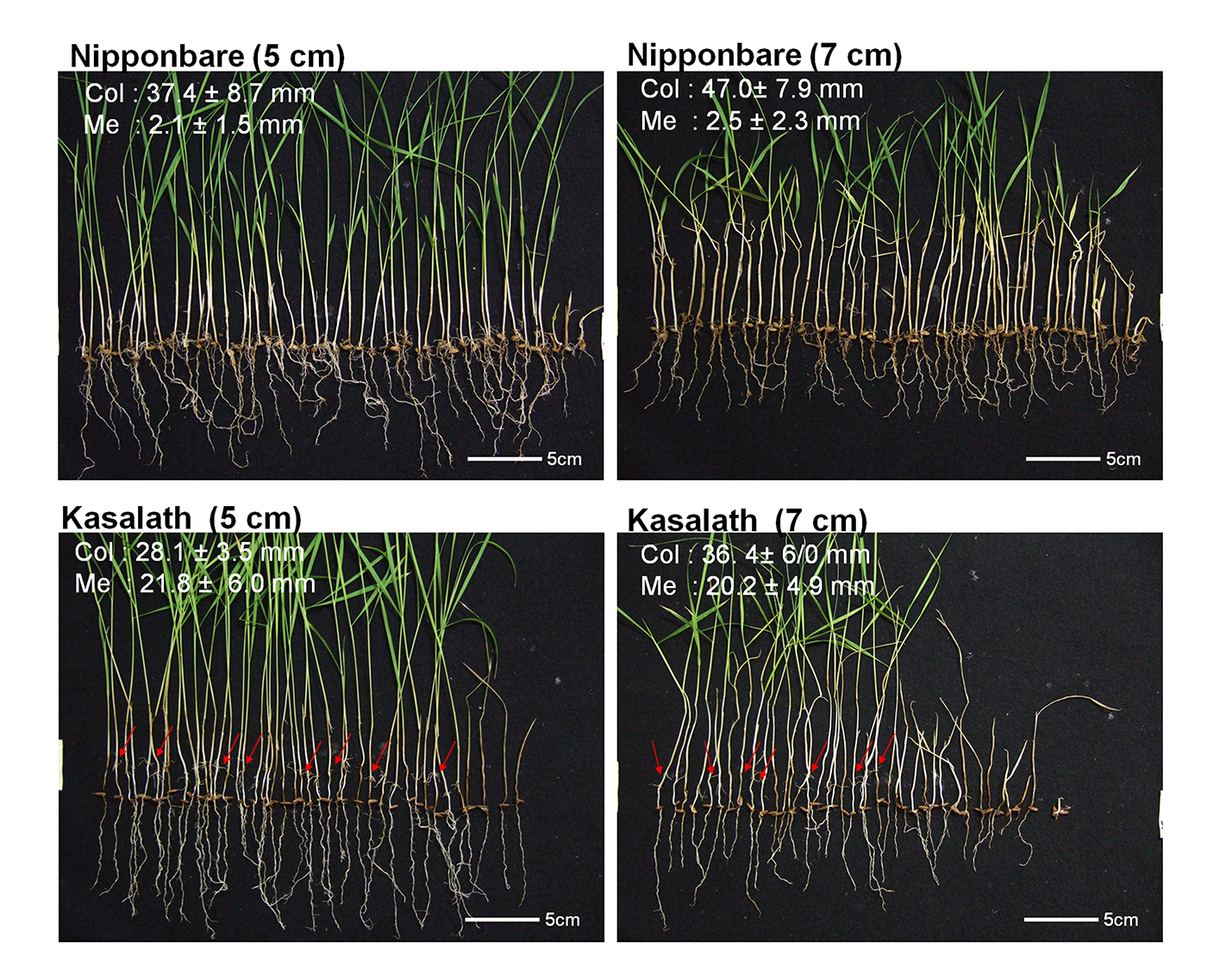

Supplement: Supplementary file 1 — Figure S1. Seedling of Nipponbare and Kasalath growing at 5 cm and 7 cm soil depth condition; 50 seeds of Nipponbare and Kasalath were sown at 5 cm and 7 cm soil depth and incubated at alternate temperatures of 30 °C and 26 °C (14 h/10 h). At 21 days after sowing, the seedling were excavated and the length of coleoptile (Col) and mesocotyl (Me) were measured. Arrows indicate mesocotyl. (TIFF 6962 kb) [file 12284_2017_173_MOESM1_ESM.tif]

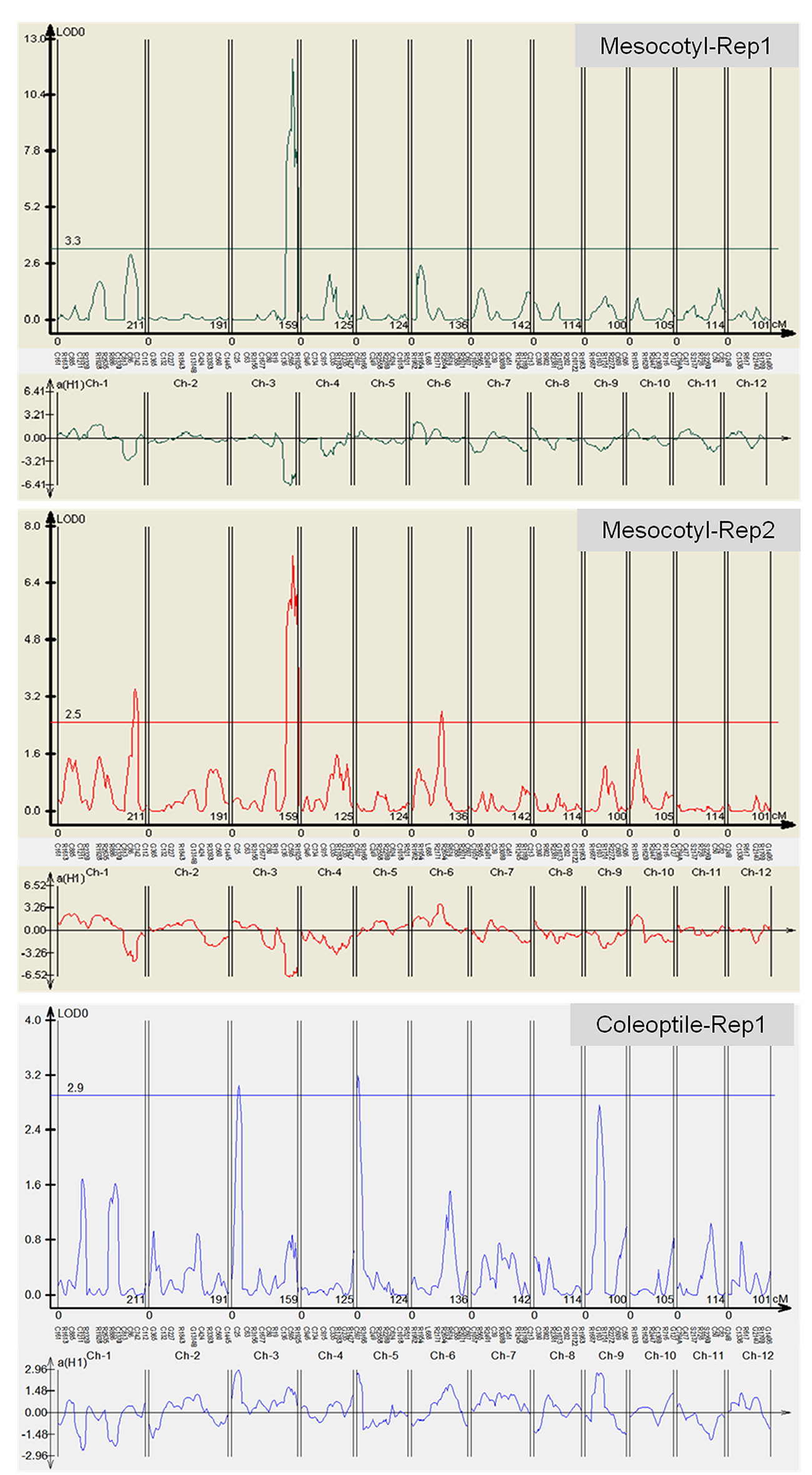

Supplement: Supplementary file 3 — Figure S2. QTL cartographer LOD peak for mesocotyl and coleoptile length at 5 cm soil depth; A QTL Cartographer plot obtained following composite interval mapping (CIM) using 2 replicates (Rep1 and 2). Significance of QTL is indicated by LOD score above the threshold values determined by permutation analysis at a significant level of P < 0.05. The graph below shows the additive effects for each of the QTL identified. Marker designations are given at the bottom and the genetic distances (cM) are given above the horizontal line. (TIFF 4914 kb) [file 12284_2017_173_MOESM3_ESM.tif]
