# Supplementary material for: Mesocotyl Elongation is Essential for Seedling Emergence Under Deep-Seeding Condition in Rice
Source: Rice (N Y). 2017 Jul 14;10:32. doi: 10.1186/s12284-017-0173-2 (PMC5511125; doi:10.1186/s12284-017-0173-2)
Supplement: Supplementary file 2 — Table S1. Variation of the mesocotyl and coleoptile length for 57 rice accessions from RDRS collection. (PDF 73 kb) [file 12284_2017_173_MOESM2_ESM.pdf]

Supplementary Table 1. Variation of the mesocotyl and coleoptile length for 57 rice accessions from RDRS collection

No.	Name	WRC No.[a]	Origin	Variety group	Mesocotyl length (mm)		Coleoptile length (mm)	
					Mean	S.D	Mean	S.D
1	NIPPONBARE	WRC 01	JAPAN	*Japonica*	3.2	± 2.5	43.0	± 5.6
2	KASALATH	WRC 02	INDIA	*Indica I*	36.3	± 3.2	18.9	± 4.1
3	BEI KHE	WRC 03	CAMBODIA	*Indica II*	4.8	± 3.9	32.2	± 4.9
4	JENA 035	WRC 04	NEPAL	*Indica I*	26.5	± 6.6	18.2	± 3.5
5	NABA	WRC 05	INDIA	*Indica II*	12.9	± 4.5	29.8	± 5.0
6	PULUIK ARANG	WRC 06	INDONESIA	*Indica II*	7.7	± 4.0	26.8	± 6.6
7	DAVAO 1	WRC 07	PHILIPPINES	*Indica II*	1.2	± 0.5	23.3	± 5.2
8	RYOU SUISAN KOUMAI	WRC 09	CHINA	-	9.4	± 4.4	38.8	± 3.8
9	SHUUSOUSHU	WRC 10	CHINA	-	21.5	± 9.0	26.9	± 7.2
10	JINGUOYIN	WRC 11	CHINA	*Indica II*	2.4	± 1.0	34.2	± 7.1
11	ASU	WRC 13	BHUTAN	-	3.9	± 1.6	39.3	± 2.1
12	IR 58	WRC 14	PHILIPPINES	*Indica II*	6.6	± 2.2	29.1	± 5.5
13	CO 13	WRC 15	INDIA	*Indica II*	1.5	± 0.6	37.2	± 3.0
14	KEIBOBA	WRC 17	CHINA	*Indica II*	4.9	± 2.8	37.4	± 5.1
15	QINGYU(SEIYU)	WRC 18	TAIWAN	*Indica II*	9.0	± 2.7	35.5	± 3.8
16	DENG PAO ZHAI	WRC 19	CHINA	*Indica II*	15.9	± 2.2	34.8	± 2.7
17	TADUKAN	WRC 20	PHILIPPINES	*Indica II*	0.3	± 0.4	35.5	± 7.9
18	SHWE NANG GYI	WRC 21	MYANMAR(BURM	*Indica II*	5.3	± 3.2	37.1	± 6.0
19	CALOTOC	WRC 22	PHILIPPINES	*Indica I*	10.6	± 8.0	37.4	± 6.8
20	LEBED	WRC 23	PHILIPPINES	-	18.1	± 8.2	30.3	± 6.2
21	PINULUPOT 1	WRC 24	PHILIPPINES	-	2.3	± 0.4	38.9	± 4.3
22	MUHA	WRC 25	INDIA	*Indica I*	33.6	± 7.9	23.3	± 4.6
23	JHONA 2	WRC 26	INDIA	*Indica I*	18.8	± 7.9	32.4	± 6.4
24	NEPAL 8	WRC 27	NEPAL	*Indica I*	33.8	± 5.0	20.8	± 2.6
25	JARJAN	WRC 28	BHUTAN	*Indica I*	37.0	± 2.8	19.9	± 1.6
26	KALO DHAN	WRC 29	NEPAL	*Indica I*	31.5	± 5.0	17.3	± 3.0
27	ANJANA DHAN	WRC 30	NEPAL	*Indica I*	25.5	± 14.4	22.8	± 8.4
28	SHONI	WRC 31	BANGLADESH	*Indica I*	19.8	± 7.7	28.7	± 4.2
29	TUPA 121-3	WRC 32	BANGLADESH	*Indica I*	31.6	± 5.9	21.3	± 2.9
30	SURJAMUKHI	WRC 33	INDIA	*Indica I*	22.8	± 4.4	25.4	± 2.6
31	ARC 7291	WRC 34	INDIA	*Indica I*	29.1	± 5.9	22.3	± 4.3
32	ARC 5955	WRC 35	INDIA	*Indica I*	27.0	± 7.8	25.1	± 7.6
33	RATUL	WRC 36	INDIA	*Indica I*	28.1	± 8.3	19.1	± 6.6
34	ARC 7047	WRC 37	INDIA	*Indica I*	31.8	± 5.3	23.2	± 6.1
35	ARC 11094	WRC 38	INDIA	-	16.5	± 6.3	30.0	± 5.3
36	BADARI DHAN	WRC 39	NEPAL	*Indica I*	29.7	± 4.7	23.3	± 4.8
37	NEPAL 555	WRC 40	INDIA	*Indica I*	31.3	± 7.2	22.2	± 6.8
38	KALUHEENATI	WRC 41	SRILANKA	*Indica I*	1.3	± 0.7	24.3	± 6.8
39	LOCAL BASMATI	WRC 42	INDIA	*Indica I*	29.2	± 11.3	19.5	± 5.2
40	DIANYU 1	WRC 43	CHINA	*Japonica*	2.8	± 1.6	45.1	± 3.4
41	BASILANON	WRC 44	PHILIPPINES	*Indica I*	35.2	± 3.7	19.0	± 3.6
42	MA SHO	WRC 45	MYANMAR(BURM	*Tropical Japonica*	25.6	± 3.2	27.7	± 3.7
43	KHAO NOK	WRC 46	LAOS	*Tropical Japonica*	23.8	± 5.5	26.6	± 4.6
44	JAGUARY	WRC 47	BRAZIL	*Tropical Japonica*	7.7	± 4.7	31.4	± 5.4
45	KHAU MAC KHO	WRC 48	VIETNAM	*Tropical Japonica*	24.4	± 8.0	28.6	± 7.7
46	PADI PERAK	WRC 49	INDONESIA	*Tropical Japonica*	28.9	± 8.4	21.4	± 3.6
47	REXMONT	WRC 50	USA	*Tropical Japonica*	2.7	± 0.7	25.0	± 2.0
48	URASAN 1	WRC 51	JAPAN	*Tropical Japonica*	4.5	± 4.7	27.5	± 5.1
49	KHAU TAN CHIEM	WRC 52	VIETNAM	*Tropical Japonica*	4.4	± 2.2	32.7	± 6.1
50	TIMA	WRC 53	BHUTAN	*Tropical Japonica*	22.0	± 4.8	31.6	± 3.7
51	TUPA 729	WRC 55	BANGLADESH	*Tropical Japonica*	8.2	± 2.3	49.2	± 2.8
52	MILYANG 23	WRC 57	REP.KOREA	*Indica II*	11.3	± 7.8	32.2	± 5.5
53	RADIN GOI SESAT	WRC 61	MALAYSIA	*Indica II*	4.6	± 1.6	27.0	± 2.7
54	BLEIYO	WRC 63	THAILAND	*Indica II*	25.1	± 5.2	22.7	± 4.8
55	RAMBHOG	WRC 65	INDONESIA	*Indica II*	15.7	± 8.3	32.9	± 4.2
56	PHULBA	WRC 67	INDIA	*Japonica*	5.9	± 3.5	40.2	± 4.3
57	KHAO NAM JEN	WRC 68	LAOS	*Japonica*	11.8	± 9.8	39.2	± 9.1

[a] World rice collection (WRC) number corresponds to the accession number of RDRS at the NIAS Gene bank (Kojima et al. 2005).
